# Descriptive analysis of horse movement networks during the 2015 equestrian season in Ontario, Canada

**DOI:** 10.1371/journal.pone.0219771

**Published:** 2019-07-11

**Authors:** Kelsey L. Spence, Terri L. O’Sullivan, Zvonimir Poljak, Amy L. Greer

**Affiliations:** Department of Population Medicine, Ontario Veterinary College, University of Guelph, Guelph, Ontario, Canada; University of Illinois College of Veterinary Medicine, UNITED STATES

## Abstract

Horses are a highly mobile population, with many travelling locally, nationally, and internationally to participate in shows and sporting events. However, the nature and extent of these movements, as well as the potential impact they may have on disease introduction and spread, is not well documented. The objective of this study was to characterise the movement network of a sample of horses in Ontario, Canada, over a 7-month equestrian season. Horse owners (n = 141) documented their travel patterns with their horse(s) (n = 330) by completing monthly online questionnaires between May and November 2015. Directed networks were constructed to represent horse movements in 1-month time periods. A total of 1754 horse movements met the inclusion criteria for analysis. A variety of location types were included in each monthly network, with many including non-facilities such as parks, trails, and private farms. Only 34.3% of competitions attended by participants during the study period were regulated by an official equestrian organisation. Comparisons of the similarity between monthly networks indicated that participants did not travel to the same locations each month, and the most connected locations varied between consecutive months. While the findings should not be generalized to the wider horse population, they have provided greater insight into the nature and extent of observed horse movement patterns. The results support the need to better understand the variety of locations to which horses can travel in Ontario, as different types of locations may have different associated risks of disease introduction and spread.

## Introduction

The movement of livestock is one of the key drivers of infectious disease introduction and spread. The availability of comprehensive animal movement databases has advanced the understanding of epidemic risk in livestock populations, including those of cattle [1–6], pigs [7–10], and sheep [11,12]. While many countries have traceability requirements for livestock, similar programs are often difficult to implement in horse populations. Previous explorations of horse movements have demonstrated high variability among horse use, the types of facilities in which they live, frequency of their movements, and reasons for travel [13–21]. In some countries, horses are included in traceability requirements and therefore their characteristics, movements, and time-varying spatial locations can be described [15,16,22]. In countries without these requirements, description of horse populations have been gathered through questionnaire-based methods [13,14,17–19]. While horses are defined as livestock under the *Livestock Identification Act* in Ontario, Canada, they are often regarded as pets or companions by their owners. Furthermore, horses are not included in any traceability requirements, limiting descriptions of the Ontario horse population to questionnaire-based research [20,23] and the Census of Agriculture (conducted every five years).

Despite the limited data published on the Canadian horse population, the industry represents a valuable component of the economy with an estimated contribution of over CA$19 billion in 2010 [24]. Horses within the industry are highly mobile, travelling locally, nationally, and internationally to participate in shows and sporting events [20]. However, the nature and extent of these movements, as well as the potential impact they may have on disease introduction and spread, is not well understood. Significant outbreaks, such as the 2007 equine influenza outbreak in Australia [25–27] and the 2011 equine herpes virus outbreak in Utah [28], have highlighted the potential risk of disease spread as a result of horse movements between locations. In order to better estimate the potential impact of horse movements on disease spread, network analysis can be used to describe and characterise movement patterns [15,16,21,22]. In particular, knowledge of the nature and extent of horse movement patterns can indicate areas to target for disease prevention and control.

The objective of this study was to characterise the observed movement network of a sample of horses in Ontario over a 7-month period (May to November 2015). The aim of the study was not to generalise the movement patterns to the wider horse population, but rather to explore the observed movement network as an initial phase of understanding patterns of movements that might be made by horses in Ontario. This study follows upon the previous work of Spence and colleagues [20] in which the authors provide a descriptive account of the demographics and movement frequencies of the sample of horses included in this current study.

## Materials and methods

This study was reviewed and approved by the University of Guelph Research Ethics Board (REB #15FE013). Written consent was obtained by participants involved in this study. All data were anonymised prior to conducting the analysis reported in this study. Details of participant recruitment, data collection, and horse characteristics have been previously described [20]. A brief overview of the longitudinal study is provided herein.

### Study population

A longitudinal study was conducted to collect horse movement data from a convenience sample of horse owners in Ontario from May to November 2015. Given the absence of an available registry of horses, owners, or facilities, a sampling frame could not be established for recruitment and sample size calculation. A variety of electronic, print, and in-person methods were used for recruitment, including the distribution of the study information through mailing lists of several equestrian associations. Therefore, the study population consisted of the horse owners that responded to an invitation to participate in the longitudinal study. At the time of enrolment, horse owners provided descriptive characteristics of their horse(s) and the facility where their horse(s) spent the majority of their time (referred to as their ‘home facility’). Participants could choose to enrol up to 10 horses if they were the person responsible for those horses.

### Horse movement data

Detailed horse movement data were collected from participants on a monthly basis between May and November using an online questionnaire [20]. Data describing horse movements were entered into a relational database in Microsoft Access 2016 (Microsoft Corporation, Redmond, WA, USA). The database included: 1) the departure and return dates of each horse’s movement on/off the facility; 2) the reason for travel; 3) the city/town of the destination; 4) the name of the destination (e.g. venue name), if available; and 5) descriptive information relating to each movement (e.g. the reason for travel). A set of exclusion criteria were developed for incomplete horse movement records to maintain consistency across the dataset. For inclusion in the current study, movement records provided by participants must have included: 1) the complete dates of movement (i.e. both departure and return date, if applicable); and 2) a minimum of the city/town of the destination (i.e. participants must have provided further details beyond that their horse travelled during the month). Horses were categorised into ‘competition’ and ‘leisure’ disciplines based on their main use as reported by their owners [20]. Competition horses were those kept to compete in equestrian disciplines such as dressage, eventing, and hunter-jumper, while leisure horses were those kept for leisure riding and companionship. Movements of racehorses (i.e. horses used for Standardbred or Thoroughbred racing) were excluded from analysis due to significant participant loss to follow-up during the initial phases of the longitudinal study.

Descriptive characteristics of the locations were determined by evaluating the disciplines of horses that travelled to them each month and their reasons for travel. Categories for location type included: home facilities, competition venues, riding locations (i.e. locations where participants attended for leisure rides), training facilities (e.g. performance/training clinics or off-site lessons), healthcare (e.g. veterinary clinics, farriers, breeding), other (e.g. sales barn), and mixed use facilities. Mixed use facilities were defined as facilities that had more than one purpose throughout the month, such as those that were home facilities but also hosted competitions. In Ontario, facilities where horses are kept and/or visit are not always classified as registered businesses or farms, and therefore there is no existing standardised list of horse facilities. In addition, locations attended by horse owners can include non-facilities (e.g. parks and trails). Therefore, destination information was provided by participants through open-ended questions in the questionnaire. Publicly available records were searched to confirm all geographic locations and/or destination names provided by the participants. If a location was named by more than one participant, the name was standardised to ensure consistency throughout the dataset (e.g. ensuring that ‘OVC’ was recognised as ‘Ontario Veterinary College’). Furthermore, the city/town provided by participants were cross-referenced with the North American Atlas Population Places Dataset [29]. Locations were also described by their geographic region as defined by the Canadian agricultural census [30].

Given that a large proportion of horse movements include travel to competitions [20], additional characteristics relating to competition venues were gathered through publicly available data. Details of sanctioned competitions that occurred during the study period were obtained from Equestrian Canada [31]. Event organisers that have their competitions sanctioned must follow regulations set by the provincial and/or federal equestrian organisations, including the implementation of standard biosecurity protocols. The dates and locations of sanctioned competitions were cross-referenced with the competitions attended by participants during the study period. It was assumed that participants attended the sanctioned competition if, during the same time period, they travelled to the facility for a competition.

Descriptive summaries of the study population demographics, number of horse movements per day, the proportion of within- and between-region movements, location characteristics, and the distance travelled between locations were calculated using R version 3.5.1 [32]. Within- and between-region movements were described by aggregating the data to the region-level and determining the proportion of movements that occurred within the same region compared to a different region. The distance between locations was estimated by calculating the great-circle distance (i.e. the shortest distance on a spherical surface) between the spatial coordinates of the centroid of the city/town of the location using the ‘geosphere’ package in R [33].

### Network analysis

Monthly aggregated networks were created to represent horse movements between unique locations in each calendar month during the study period. Monthly time periods were chosen as a plausible time period that considered the incubation period and time to detection of several equine pathogens of interest (e.g. equine influenza virus and equine herpes virus). Networks consisted of active nodes (locations that had at least one movement) and edges (the movement of at least one horse) within the monthly time period. Edges were directed to represent the direction of movement between a source and destination. Because horse movements are often bidirectional (i.e. horses return to their home facility after travelling to a destination), two directed edges that represented the outgoing and incoming movement of the horse(s) could exists between dyads. Edges were unweighted as the number of horses moved between two locations was not considered. Several network measures were calculated to characterise movements within each monthly time period:

1. Network size: the total number of nodes and edges [34].
2. Reciprocity: the proportion of reciprocal edges in the network [35]. Reciprocity ranges from 0 to 1, where a value of 1 indicates that all edges are reciprocal.
3. Assortativity: a measure of whether nodes tend to be connected to similar nodes. A value of 1 indicates that nodes tend to be connected to similar nodes, and a value of -1 indicates that nodes tend to be connected to dissimilar nodes [36]. Assortativity was calculated based on three node attributes: degree, type, and discipline.
4. Giant strong component (GSC) size: the largest set of locations that are connected to each other through directed edges, either directly or indirectly [37].
5. Loyalty: the proportion of preserved direct contacts of a location between two consecutive months, given by the Jaccard index [38]. Loyalty ranges from 0 to 1, where 1 means that a horse from a given location travelled to the same set of locations in consecutive months.
6. In-degree: for a given node, the in-degree is the number of locations from which horses were received [37].
7. Out-degree: for a given node, the out-degree is the number of locations to which horses were transported [37].

Highly connected nodes were further assessed using the similarity index to determine whether the same nodes were also highly connected in consecutive monthly networks [39]. Locations were ranked by their average degree (the average of the node’s in-degree and out-degree) in each monthly network. Highly connected nodes were defined as those within the top 10% of values according to their average degree [39]. The proportion of locations that retained their highly connected position in subsequent monthly networks was given by the Jaccard index. All network analyses were conducted in R using the ‘igraph’ [40] package.

### Data completeness

Because the longitudinal study required participants to complete a questionnaire each month, variation was observed in the response rate between months [20]. The impact of missing data due to participant non-response in select months of the longitudinal study was evaluated by repeating the network analyses at alternate levels of data completeness. Three data completeness scenarios were evaluated: 1) the ‘any’ scenario, where participants were included if they completed at least one out of seven monthly questionnaires (n = 197 participants); 2) the ‘most’ scenario, where participants must have completed any five out of seven monthly questionnaires (n = 141 participants); and 3) the ‘all’ scenario, where participants must have completed all seven monthly questionnaires (n = 87 participants).

## Results

Comparisons between the data completeness scenarios suggested that the networks did not vary substantially between scenarios. However, trends in the number of nodes and edges in the networks were most pronounced once the majority of participants who were lost to follow-up were excluded. Therefore, the results of the ‘most’ data completeness scenario (i.e. movement data from participants with at least five surveys completed during the longitudinal study) are discussed herein. Results for the ‘any’ (199 participants) and ‘all’ (87 participants) data completeness scenarios are further described in the supporting information (S2 File).

### Study population

The study population consisted of 141 horse owners who provided information for 330 horses. Owners enrolled a median of 1 horse into the study [interquartile range (IQR) 1–3]. Seventy-seven percent of owners (n = 109) indicated they kept their horse(s) at home facilities shared with other owners, and 73.0% (n = 103) described their home facilities as multidisciplinary (i.e. mixture of competition and leisure horses). Of the horses included in the study, 63.6% (n = 210) were kept for competition purposes and 36.1% (n = 119) were kept for leisure purposes.

### Description of horse movements

**Fig 1:**
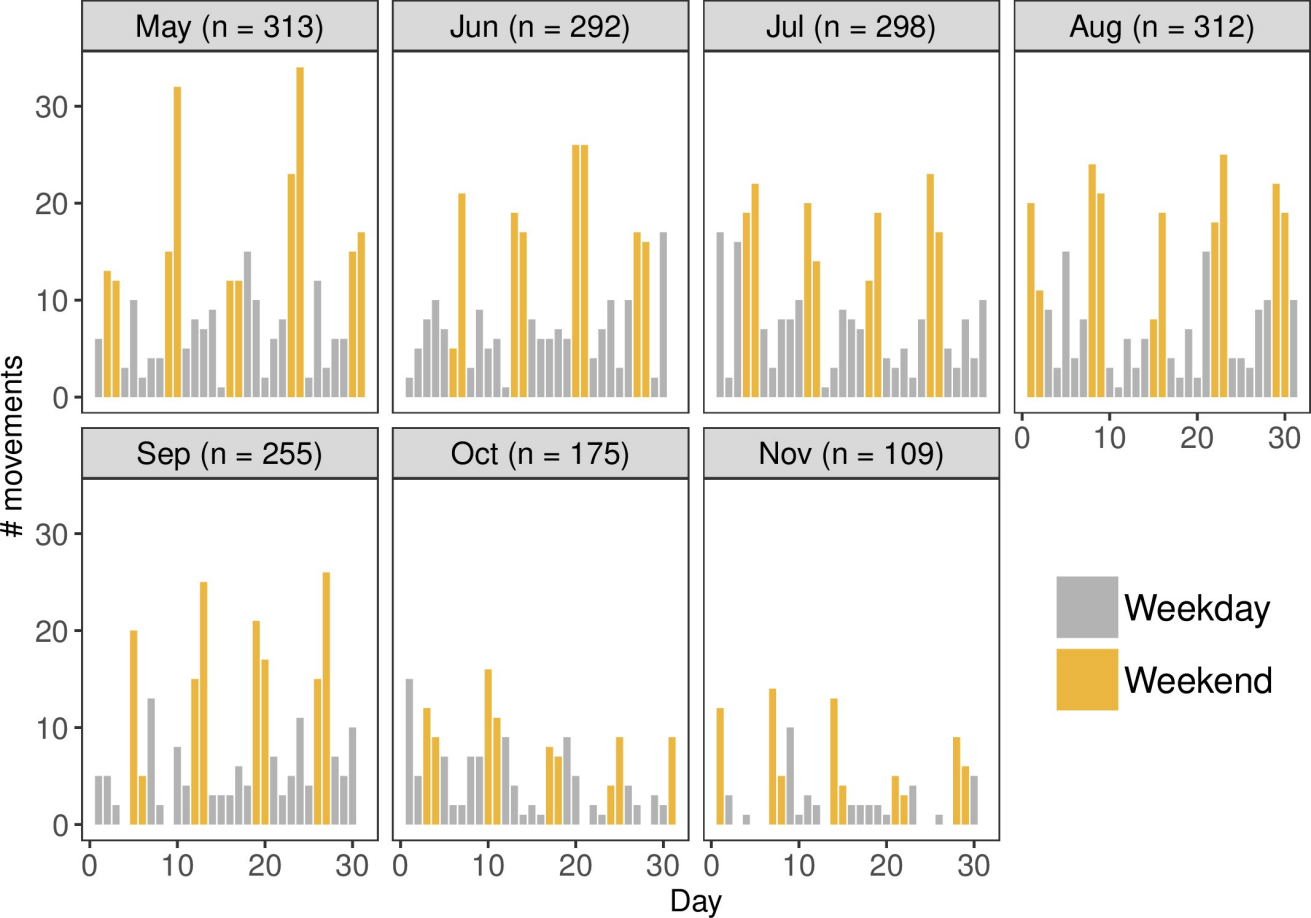
Number of daily movements of a sample of horses in Ontario, Canada between May and November 2015. The ‘n’ in each panel refers to the total number of horse movements during that month.

In total, 1754 horse movements met the inclusion criteria for further analysis. The largest number of movements occurred in May (n = 313), followed by a peak in August (n = 312) and a decline in movements until November (n = 109) (Fig 1). Daily movements during the week were less frequent, and the total number of daily movements increased on the weekends (Saturdays and Sundays).

**Fig 2:**
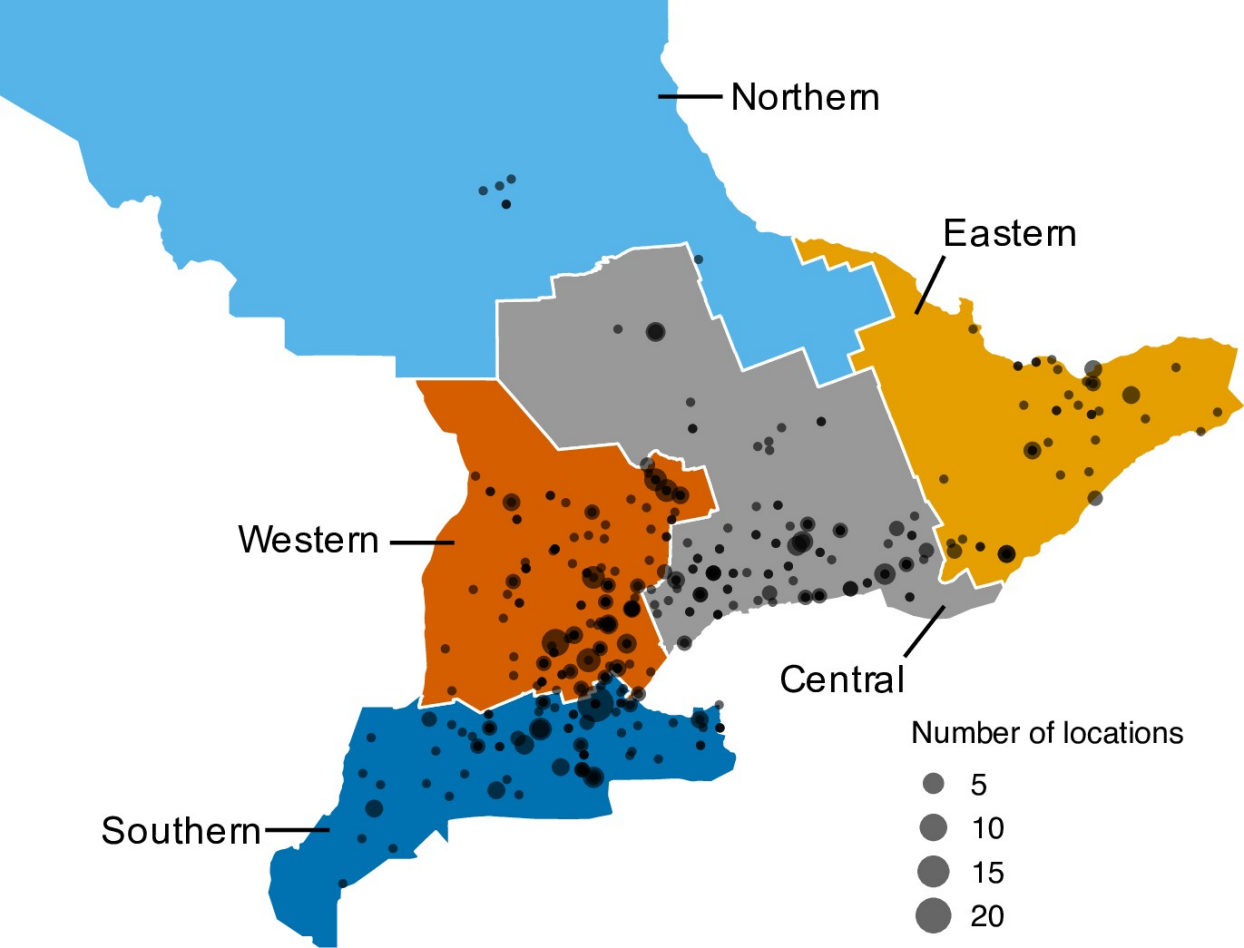
Locations in Ontario (n = 539) travelled by a sample of horses between May and November 2015. Colours represent agricultural census regions as defined by Statistics Canada. Point size represents the cumulative number of locations present in the same area.

**Fig 3:**
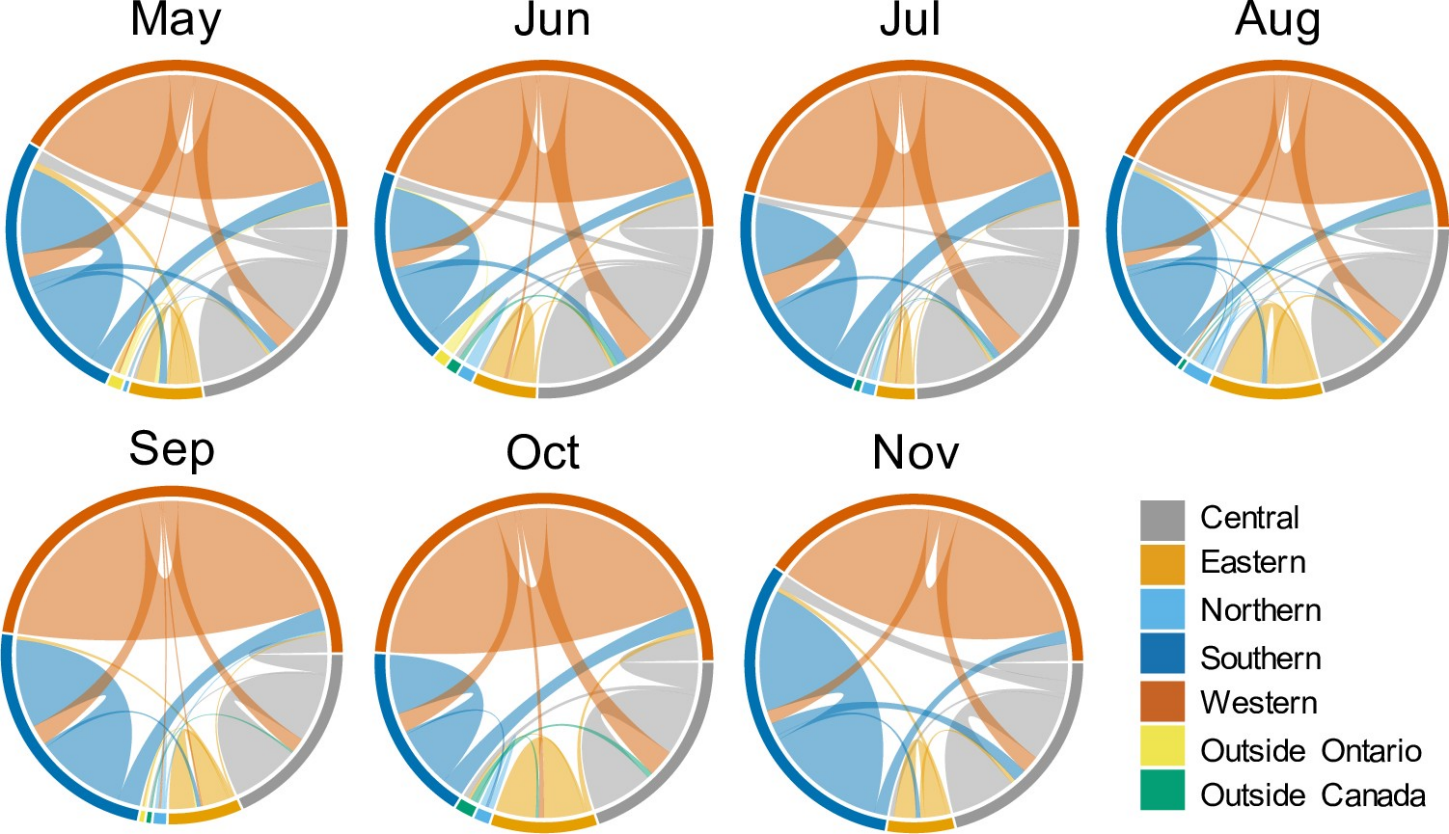
Within- and between-region movements of horses within each monthly network during the longitudinal study in 2015. Self-links represent movements within a region and links connecting two regions represents movements between regions. Central, eastern, northern, southern, and western regions refer to agricultural census regions in Ontario (see Fig 1 for defined areas).

There were 553 unique locations attended by horses over the entire study period. Of those, 539 locations (97%) were in Ontario (Fig 2). Seven locations were attended outside of Ontario (i.e. movements to different provinces), and seven locations were attended internationally (to the United States of America). The median distance between two locations during the study period was 47 km (IQR 21–88 km). The distribution of distances between pairs of locations was right-skewed, with a large number of movements covering short distances, and a small number covering long distances (up to 3214 km) (Table 1). Over the entire study period, 73% of horse movements occurred within the same geographic region, compared to 27% that occurred between geographic regions. The highest proportion of between-region movements occurred in May (32%), compared to the lowest proportion of between-region movements which occurred in September (21%) (Fig 3).

**Table 1:** Median distance (in kilometres) between pairs of locations visited by a sample of horses in Ontario, Canada between May and November 2015.

Month	Minimum^a^	Median (IQR)^b^	Maximum
May	0	47 (23–81)	3214
June	0	47 (18–92)	1580
July	0	42 (18–81)	767
August	0	54 (24–88)	465
September	0	54 (27–87)	464
October	0	47 (28–107)	1096
November	0	41 (22–59)	492

^a^Distance of 0 km indicates that the given pairs of locations were in the same city/town

^b^Interquartile range

Of the 553 locations, 132 (23.9%) represented participants’ home facilities (both initial home facilities and ones that they moved to during the study period). An additional 143 locations (25.9%) hosted competitions and 105 locations (19.0%) were used for riding (e.g. trails and parks). A total of 367 movements (20.9%) were to attend competitions during the study period. Only 34.3% (126/367) of movements to attend competitions were to participate in sanctioned events. Out of the 143 locations that hosted competitions during the study period, 29 (20.3%) hosted exclusively sanctioned events, while 11 (7.69%) hosted both sanctioned and unsanctioned events.

### Network analysis

**Fig 4:**
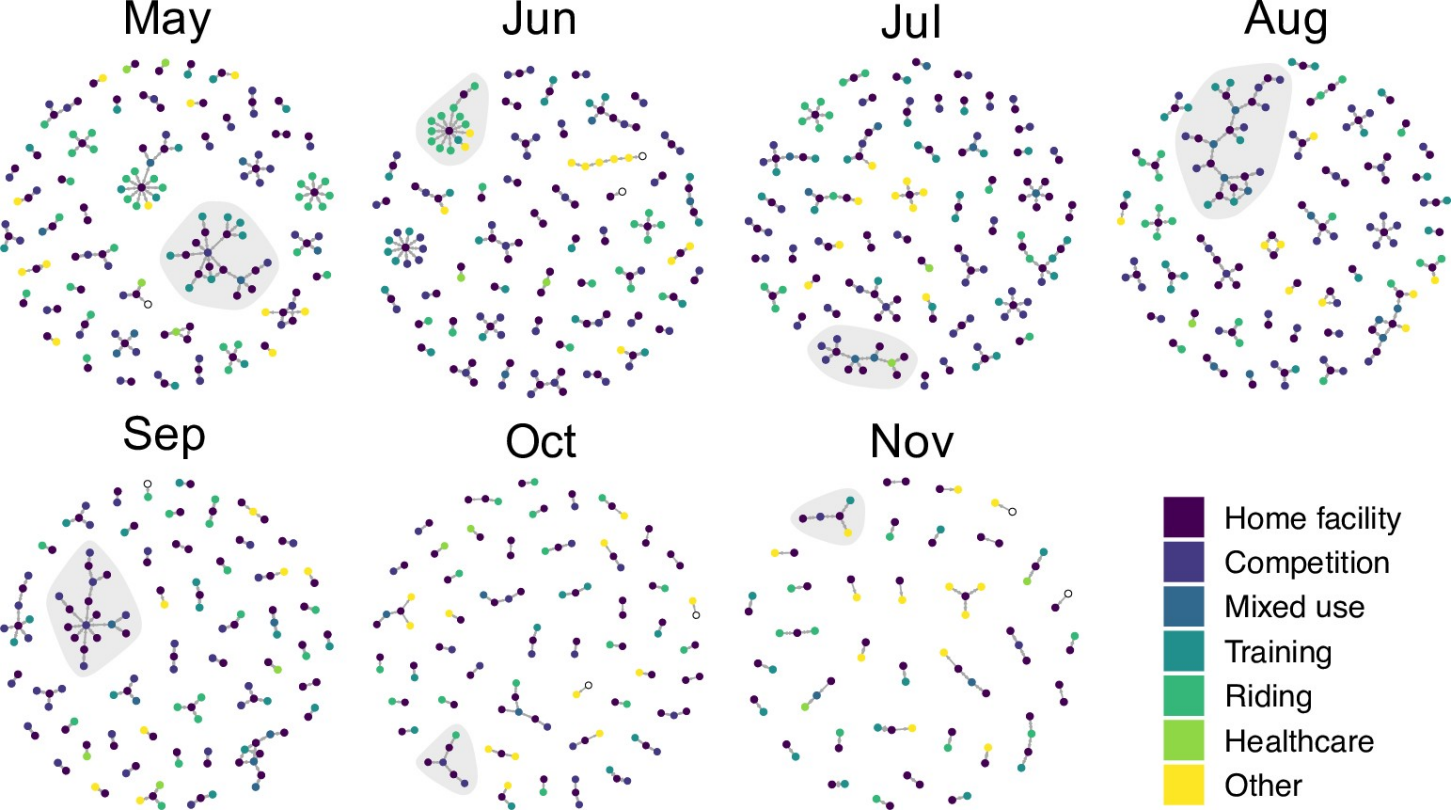
Monthly networks of horse movements between May and November 2015. Nodes represent locations and edges represent the directed movement(s) of at least one horse during the month. Node colour represents the type of location as determined by the reason for travelling there. The giant strong component (GSC) of each network is highlighted in grey.

**Fig 5:**
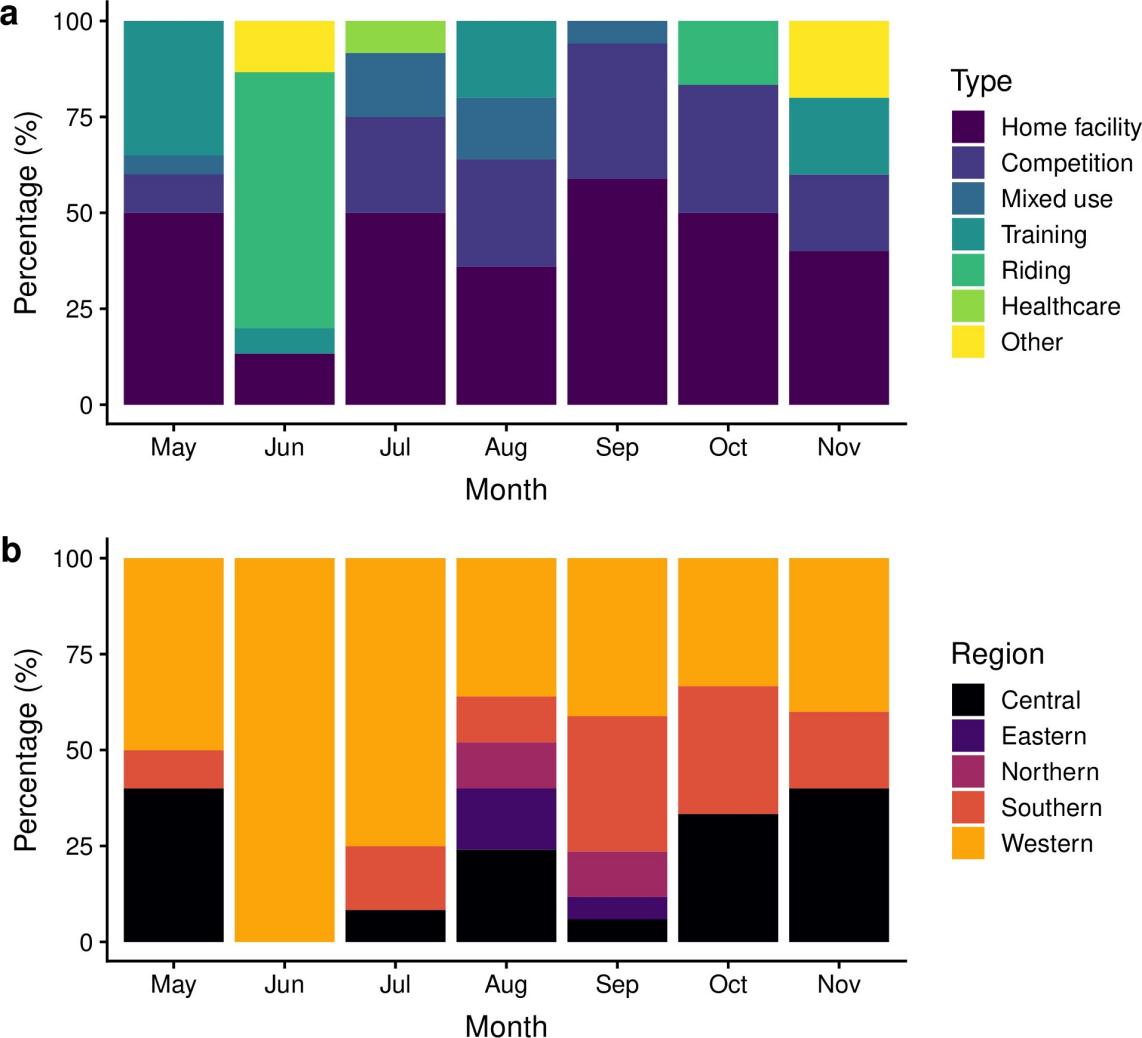
Characteristics of nodes included in the giant strong component (GSC) in each monthly network. Panels represent the (a) location type and (b) region of the nodes.

Sociograms of the monthly networks are presented in Fig 4. The number of nodes and edges in each network varied by month and decreased over the study period (Table 2). The median reciprocity was 0.96 (IQR 0.93–0.97), meaning that 96% of outgoing horse movements between two locations also had a second incoming movement (i.e. horses returned to their initial home facility). Assortativity calculations indicated a dissimilar mixing pattern by location degree, type and discipline across the study period (Table 2). Nodes in the monthly networks exhibited low in-degree and out-degree values, with nodes having a median of 1 ingoing and 1 outgoing connection (Table 2). The median GSC size across the study period was 15 nodes and ranged between 5 nodes (in November) and 20 nodes (in May) (Fig 4). Components within each network contained a mixture of location types, with several types of locations included in the GSC (Fig 5). While all monthly GSCs contained home facilities, they also contained competitions, riding locations, mixed use facilities, and healthcare services.

**Table 2:** Descriptive measures calculated for aggregated monthly networks of horse movements between May and November 2015.

Measure	Network						
	May	Jun	Jul	Aug	Sep	Oct	Nov
Nodes	171	171	165	160	144	122	84
Edges	250	234	230	247	188	139	90
Reciprocity	0.95	0.97	0.98	0.97	0.96	0.91	0.87
Assortativity (degree)	-0.26	-0.23	-0.33	-0.35	-0.07	0.19	0.03
Assortativity (type)	-0.44	-0.43	-0.42	-0.45	-0.44	-0.40	-0.38
Assortativity (discipline)	-0.12	-0.18	-0.11	-0.05	-0.14	-0.08	0.02
GSC size ^a^	20	15	12	25	17	6	5
In-degree ^b^	1 (0–10)	1 (0–12)	1 (0–5)	1 (1–5)	1 (0–8)	1 (0–4)	1 (0–3)
Out-degree ^b^	1 (0–10)	1 (0–12)	1 (0–5)	1 (0–5)	1 (0–8)	1 (0–4)	1 (0–3)

^a^ GSC = giant strong component

^b^ Median (range)

**Fig 6:**
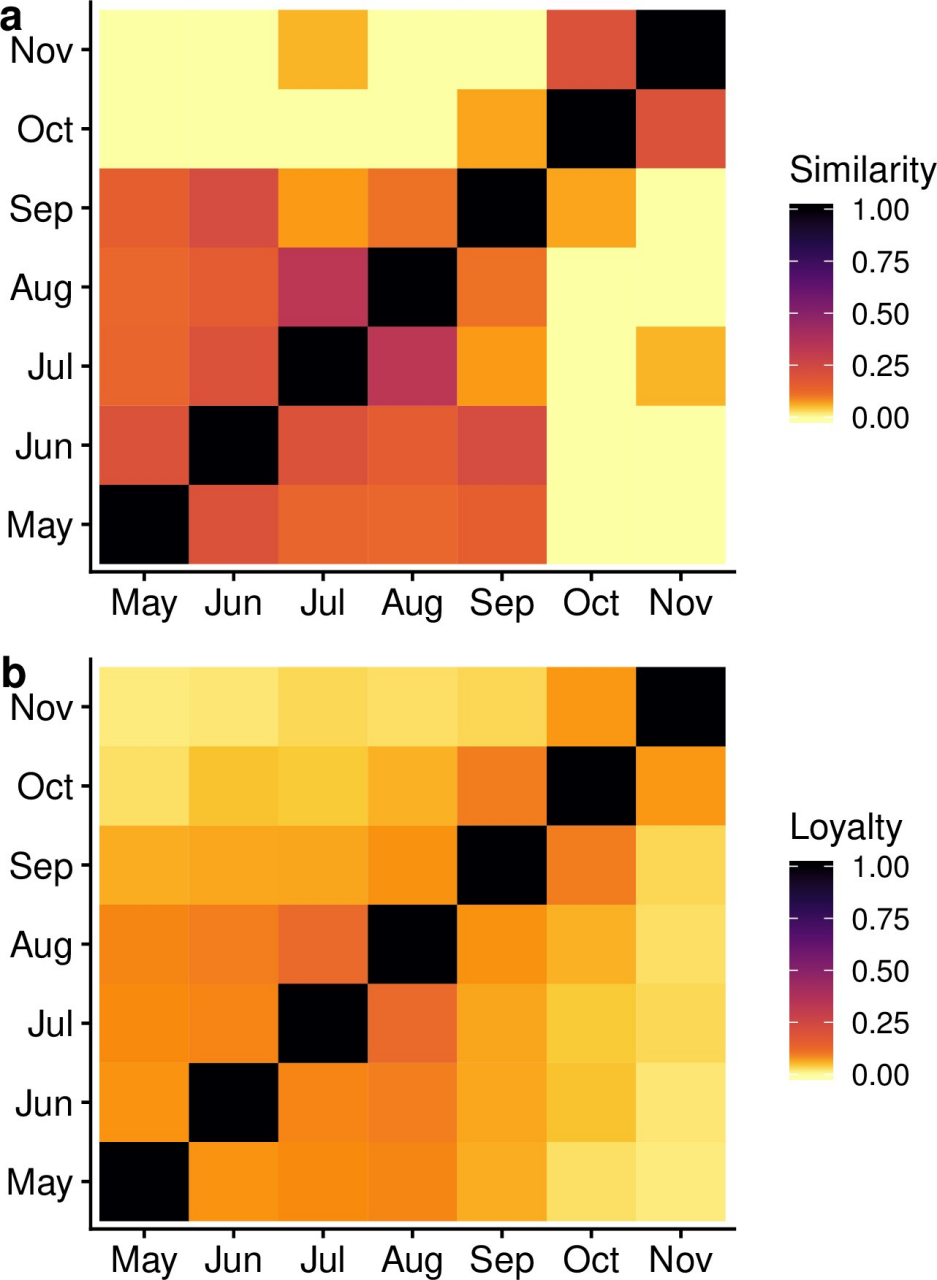
**The proportion of (a) highly connected nodes that retained their position, and (b) preserved direct contacts between locations, in consecutive monthly networks.** Highly connected nodes were defined as those within the top 10% of values according to their average degree.

Consecutive monthly network pairs exhibited low median levels of similarity and loyalty (Fig 6). The median similarity of highly connected nodes was 0.07 (IQR 0.00–0.16) when ranked by average degree (Fig 6A). Similarity between monthly networks was higher between networks closer in time compared to those farther in time, with the most similar monthly pairs being July and August (similarity = 0.33). The median loyalty across the study period was 0.066 (IQR 0.031–0.085), meaning that a median of 6.6% of preserved node neighbours occurred between the monthly networks (Fig 6B). Similar to the highly connected nodes, monthly networks that were closer in time to each other had a higher proportion of preserved neighbours compared to monthly networks further away in time. The monthly network pair with the highest proportion of preserved edges was July and August (loyalty = 0.11).

## Discussion

In this study, we characterised the movements of a sample of horses in Ontario using previously collected longitudinal movement data [20]. Horses tended to travel on weekends throughout the study period and travelled within short distances (less than 50 km) between locations. The observed monthly networks contained a diverse range of geographic regions and location types, indicating the variability among horse movement patterns. Monthly networks that were closer in time to each other were most similar in regard to loyalty (retained node neighbours across months) and similarity (retained position of highly connected nodes across months). However, the low loyalty values indicated that horses travelled to different locations each month, and the low similarity values indicated that the most connected facilities changed over time. While the aim of the study was not to generalise these movement patterns to those of the wider horse population, the findings has provided greater insight into the nature and extent of the observed movement patterns of a sample of horses.

Descriptive summaries of horse movements revealed that horse owners travel more frequently with their horse on the weekend compared to during the week. The high frequency of movements on the weekends is likely driven by the recreational purposes for which owners in this study kept their horse(s) (i.e. competition and leisure activities). This result is consistent with the demonstrated higher frequency of horse movements in populations of horses that are kept for competition or equestrian purposes [13,16,17]. As the movements of racehorses were excluded from this study due to owner non-response, further research should include specifically evaluating the movements of racehorses, as they are likely to be different than the movement of horses used for competition and leisure purposes.

Most horse movements were to locations less than 50 km away from a participant’s home facility and occurred within the same geographic region. A higher frequency of local, within-region travel suggests that potential disease spread originating at a given location would have a higher probability of being contained in that region, as the majority of contacts would be in close geographic proximity. While the majority of participants travelled locally within Ontario, there are important considerations for the small proportion of horse owners who travelled internationally. Mechanisms to ensure that potential disease spread or exposure is detected quickly at horse facilities should be in place to reduce the risk of local or international disease spread [41].

The lack of a standardised list of horse locations is complicated by the variety of locations to which horses can travel, including equestrian events, outdoor spaces, and privately-owned facilities [16,17]. Furthermore, some locations could not be standardised due to participant non-disclosure (e.g. the participant could respond to the open-ended question with the response, “my friends’ farm”). Consequently, some locations could be included more than once if multiple participants had travelled to the same location. The potential ‘double-counting’ of locations would lead to a larger number of nodes and a higher degree of fragmentation than what would be observed in the true network. While a standardised list of horse facilities, or locations which horses might visit, would be beneficial for disease surveillance or for contact-tracing during an outbreak [26], the nature of horse movements might introduce challenges for implementing such policies. For instance, many locations travelled by horses include those for riding, which are not ‘named’ facilities but instead local parks, trails, and private farms. The use of horses for companionship introduces opportunities to travel to unconventional locations, which may prove difficult for traceability programs. Furthermore, the majority of competitions attended by participants during the study period were not sanctioned by an official equestrian organisation. Organisers of unsanctioned events are not required to implement disease prevention strategies, and potentially have higher risks of disease introduction and spread associated with attendance. Thus, the unique nature of horses as companion animals should be considered when determining the most effective way to introduce traceability requirements.

While the horse movement data discussed herein does not attempt to describe the complete network of horse movements in Ontario, it provides an important foundation for better understanding the movement patterns of these horses. Limitations of using questionnaire-based data can include biases caused by missing data and non-response. Nevertheless, the longitudinal approach used to describe horse movements provides a more detailed description of long-term horse movement patterns compared to previous questionnaire-based approaches at single points in time [13,17–19,21,23]. The data completeness analysis suggested that the trends in the monthly movement patterns and calculated network measures remain consistent even at different levels of data completeness. The consistency among calculated measures lends support to the notion that the results observed in this study are not solely due to variations in participant response throughout the longitudinal study. While estimates of the size of the Ontario horse population are highly varied (between 64,536 [30] and 212,500 horses [24]), we can estimate that we have captured the movements of between 0.2 to 0.7% of the population. Given that the results produced here only describe a small sample of horses and their movements, future work should focus on obtaining more complete horse movement data to better understand the impact on disease introduction and spread.

The monthly networks of horse movements exhibited several trends over the study period. The high reciprocity of the monthly networks further demonstrated the bidirectional nature of horse movements, as most horses move temporarily between locations [13,17,42]. As previously demonstrated in other equine populations, reciprocity was higher during the summer months when horse owners are temporarily moving to attend competitions [16]. Reciprocity was lower later in the study period (October and November), when more horse owners are permanently moving to new home facilities for winter boarding [20]. The difference in network-level measures between months suggests that the spread of an infectious disease on this network would behave differently depending on the time or season of introduction. Several studies of wildlife contact networks have demonstrated the importance of seasonal behavioural factors on contact patterns; for example, the increased contact frequencies during the breeding season of raccoons [43] and variability of badger contact patterns between summer and winter [44]. Seasonality has also been observed in livestock movement and contact patterns, such as the influence of the commercial breeding season on horse movements in New Zealand [14] and the contribution of shared summer pastures to infectious disease transmission in cattle [45]. Thus, seasonality, whether commercially or environmentally driven, can be an important driver for disease dynamics. In the current study, the higher frequency of movements during the summer could be due to the lack of available horse activities during the cold winter months in Ontario. Because the horses in the study were not be followed over an entire year, further research on the potential effects of seasonality on horse movements should be explored.

The small proportion of preserved node neighbours between months suggests that individual horse owners do not travel to the same locations each month. Similar loyalty patterns have been observed in other livestock network, where farms tended to interact with different trading partners over time [1,39,46]. While the positions of highly connected nodes were most similar across the summer months, a median of only 7% of nodes were consistency highly connected across the entire study period. The low proportion of nodes that retained their highly connected position suggests that a range of different locations become important in the network depending on the activities scheduled during that month. This finding further supports the need to better understand the variety of locations to which horses can travel in Ontario, as different types of locations may have different associated risks of disease introduction and spread [16].

One limitation of using questionnaire data for network analysis is that the movements of horses not in this study are not considered. For instance, other horses boarded at the participants’ home facilities and those attending locations included in this study would affect the network measures calculated herein. Unfortunately, there is little data available that would allow for the quantification of horses that attend various locations in Ontario. The unidentified horses would affect the total size of the network and may underestimate the connectivity of the larger horse population. Thus, the calculated network measures should only be interpreted within the bounds of this sample of horses. While the networks cannot be generalised between populations, the results provide insight into the travel patterns of a sample of horses in Ontario. The findings in this study support the need for further collection and analysis of horse movement data in Ontario to better understand the risk of disease introduction and spread via horse movements.

## Conclusions

This study provides a description of the movements of a sample of Ontario horses during a 7-month period in 2015. The monthly networks of horse movements demonstrated mixing between horses as they travelled to attend locations which might be unregulated by official equestrian organisations, such as local parks, private farms, and unsanctioned competitions. The use of horses for companionship introduced opportunities to travel to unconventional locations, which may have important implications for disease risk. Thus, the unique nature of horses kept as companion animals should be considered when determining effective methods for disease prevention and control. The findings support the need to better understand the variety of locations to which horses can travel in Ontario, as different types of locations may have different associated risks of disease introduction and spread. The outcomes from this study could be used to support future research to examine and refine disease prevention, control, and surveillance strategies in the population.

## Supporting information

S1 File Anonymised movements of 476 horses in Ontario, Canada between May and November 2015. Horse movements were collected from 197 horse owners using monthly online questionnaires.
(XLSX)

S2 File Results of the comparisons between questionnaire data completeness scenarios. This file includes tables and figures summarising network analysis conducted for alternate data completeness scenarios.
(PDF)
