# Supplementary material for: Descriptive analysis of horse movement networks during the 2015 equestrian season in Ontario, Canada
Source: PLoS One. 2019 Jul 11;14(7):e0219771. doi: 10.1371/journal.pone.0219771 (PMC6622551; doi:10.1371/journal.pone.0219771)
Supplement: S2 File — This file includes tables and figures summarising network analysis conducted for alternate data completeness scenarios. (PDF) [file pone.0219771.s002.pdf]

S2 File. Results of the comparisons between questionnaire data completeness scenarios.

S1 Table. The number of nodes and edges in each monthly network of horse movements at alternate levels of data completeness during the longitudinal study in 2015.

Scenario [a]	Month	Nodes	Edges
Any	May	219	319
	June	197	274
	July	194	276
	August	173	263
	September	163	219
	October	129	147
	November	100	114
All	May	105	148
	June	110	140
	July	105	146
	August	115	164
	September	101	125
	October	82	90
	November	50	53

[a] Any = at least one survey completed during the longitudinal study; all = all seven surveys completed during the longitudinal study. Results of the ‘most’ scenario (at least 5 surveys completed) is presented in the main manuscript text.

S2 Table. Descriptive measures calculated for aggregated monthly networks of horse movements at alternate levels of data completeness during the longitudinal study in 2015.

		Scenario [a]	
**Measure**	**Month**	**Any**	**All**
In-degree [b]	May	1 (0 - 10)	1 (0 - 10)
	June	1 (0 - 12)	1 (0 - 12)
	July	1 (0 - 5)	1 (1 - 5)
	August	1 (1 - 5)	1 (1 - 4)
	September	1 (0 - 10)	1 (0 - 4)
	October	1 (0 - 4)	1 (0 - 2)
	November	1 (0 - 4)	1 (0 - 3)
Out-degree [b]	May	1 (0 - 10)	1 (0 - 10)
	June	1 (0 - 12)	1 (0 - 12)
	July	1 (0 - 5)	1 (1 - 5)
	August	1 (0 - 5)	1 (0 - 4)
	September	1 (0 - 10)	1 (0 - 4)
	October	1 (0 - 4)	1 (0 - 3)
	November	1 (0 - 4)	1 (0 - 3)
Reciprocity	May	0.95	0.95
	June	0.97	0.94
	July	0.99	1.00
	August	0.97	0.98
	September	0.96	0.98
	October	0.91	0.91
	November	0.89	0.87
Assortativity (degree)	May	-0.18	-0.27
	June	-0.24	-0.17
	July	-0.37	-0.33
	August	-0.33	-0.39

	September	-0.16	-0.14
	October	0.18	-0.14
	November	0.09	-0.07
Assortativity (type)	May	-0.44	-0.45
	June	-0.43	-0.39
	July	-0.42	-0.42
	August	-0.45	-0.46
	September	-0.44	-0.45
	October	-0.40	-0.40
	November	-0.38	-0.40
Assortativity (discipline)	May	-0.11	-0.21
	June	-0.20	-0.30
	July	-0.13	-0.02
	August	-0.09	-0.15
	September	-0.14	-0.28
	October	-0.16	-0.22
	November	-0.12	-0.11
GSC size [c]	May	42	15
	June	15	15
	July	12	9
	August	25	13
	September	19	7
	October	6	4
	November	7	4

[a] Any = at least one survey completed during the longitudinal study; all = all seven surveys completed during the longitudinal study. Results of the 'most' scenario (at least 5 surveys completed) is presented in the main manuscript text.

[b] Median (range)

[c] GSC = giant strong component

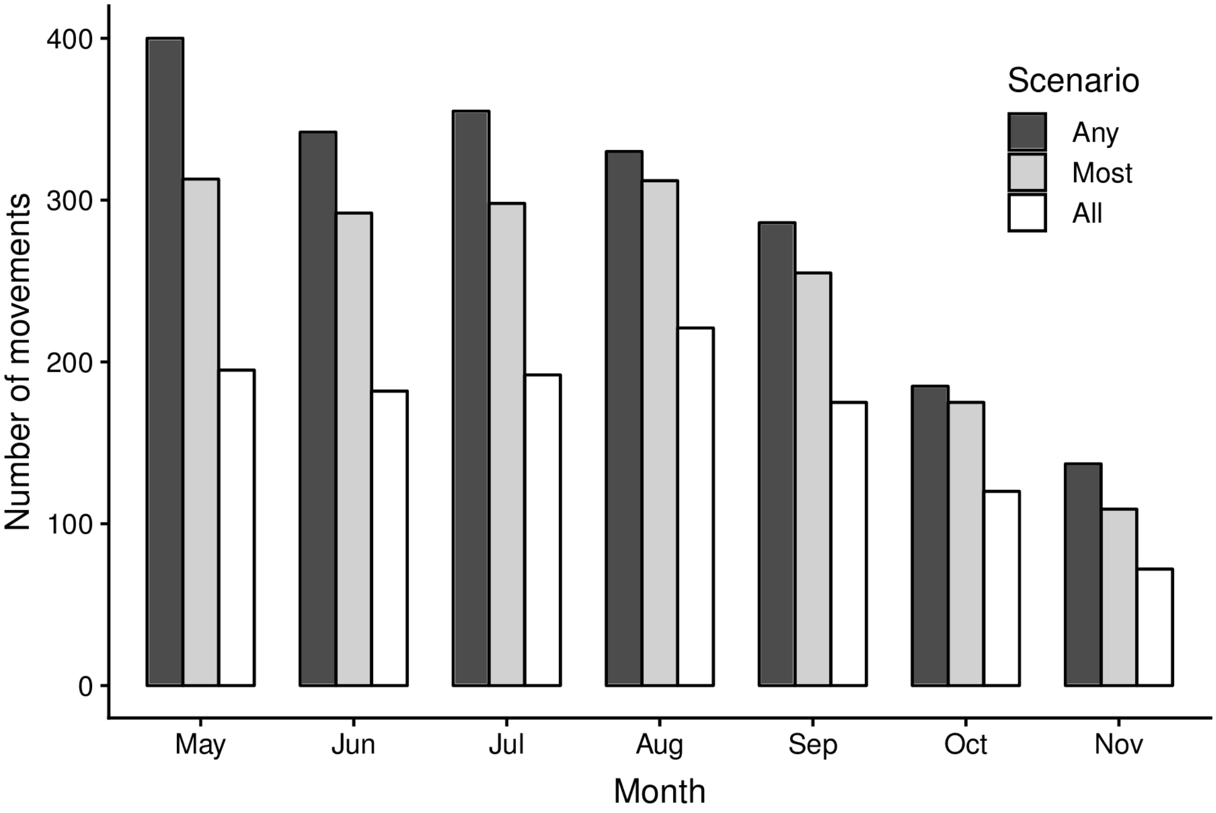

S1 Fig. Number of monthly horse movements included in each data completeness scenario. The 'any' scenario included participants with at least one survey completed during the longitudinal study (n = 2035 movements). The 'most' scenario' included participants with at least five surveys completed during the longitudinal study (n = 1754 movements). The 'all' scenario included participants with all seven surveys completed during the longitudinal study (n = 1157 movements).

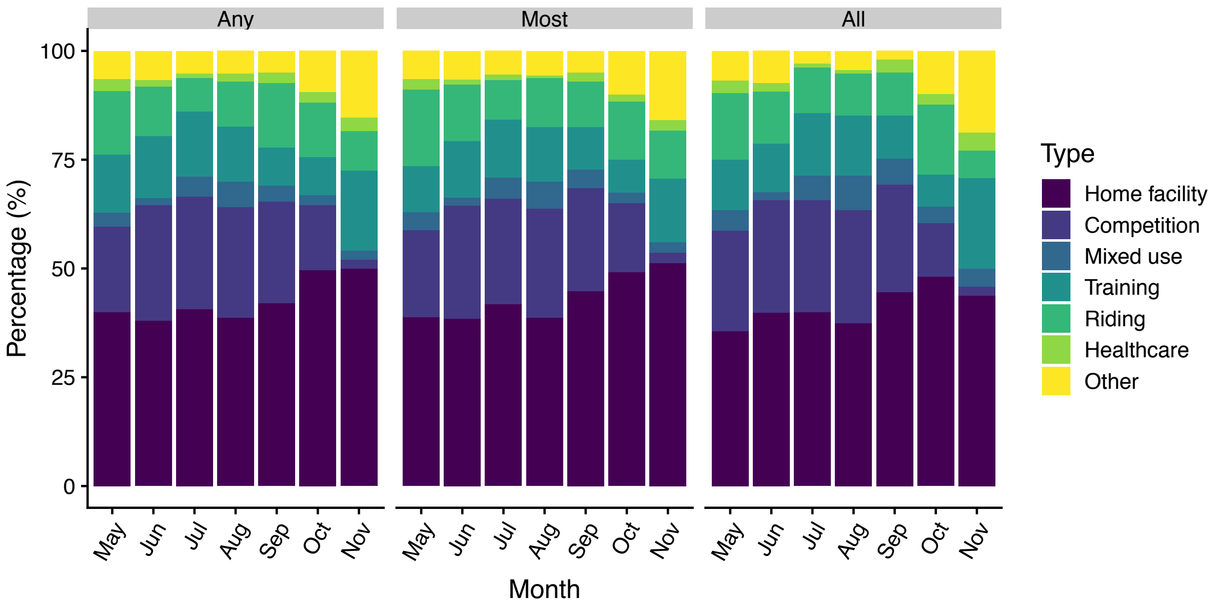

S2 Fig. Types of nodes (locations) included in monthly horse movement networks in each data completeness scenario. Scenarios: 'any' = at least one survey completed; 'most' = at least five surveys completed, 'all' = all seven surveys completed.

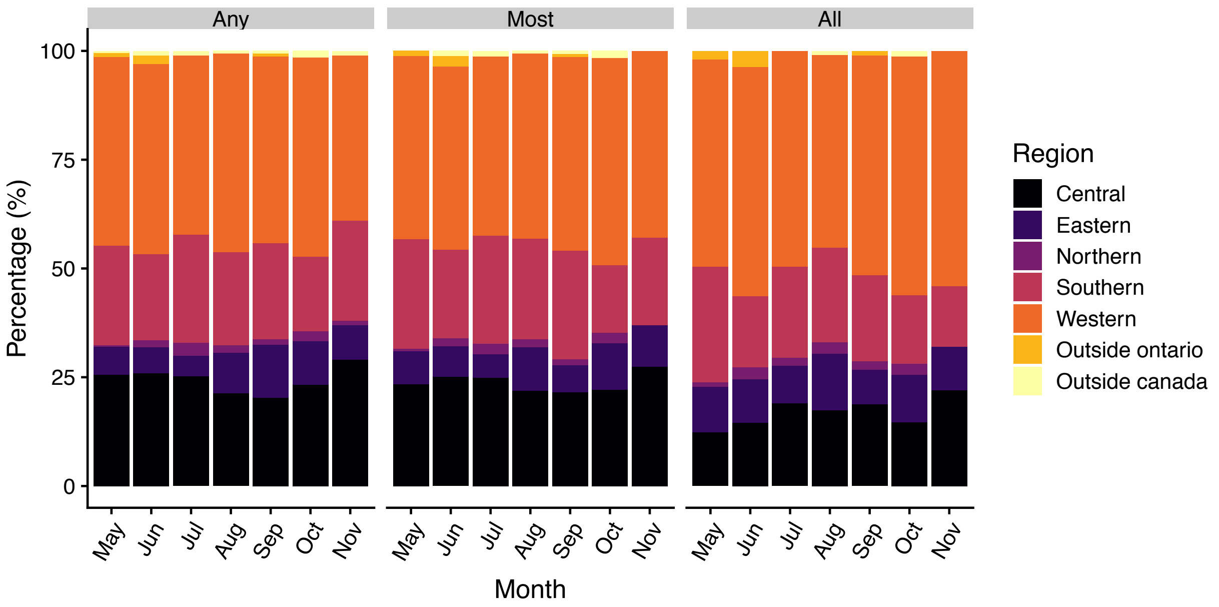

S3 Fig. Geographic region of nodes (locations) included in monthly horse movement networks in each data completeness scenario. Scenarios: 'any' = at least one survey completed; 'most' = at least five surveys completed, 'all' = all seven surveys completed. Central, eastern, northern, southern, and western regions refer to agricultural census regions in Ontario (see Fig 1 in manuscript for defined areas).

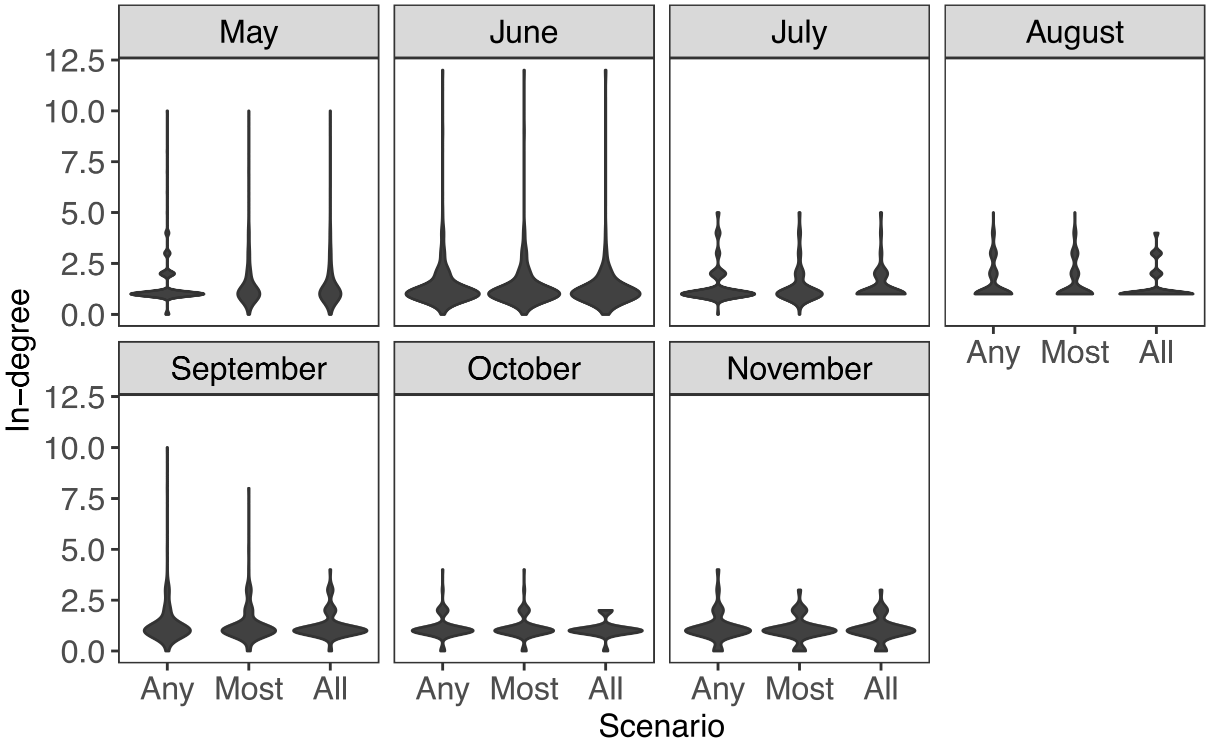

S4 Fig. In-degree distribution for monthly horse movement networks in each data completeness scenario. Each violin plot represents a kernel density estimation of the in-degree of nodes included in each network. Scenarios: 'any' = at least one survey completed; 'most' = at least five surveys completed; 'all' = all seven surveys completed.

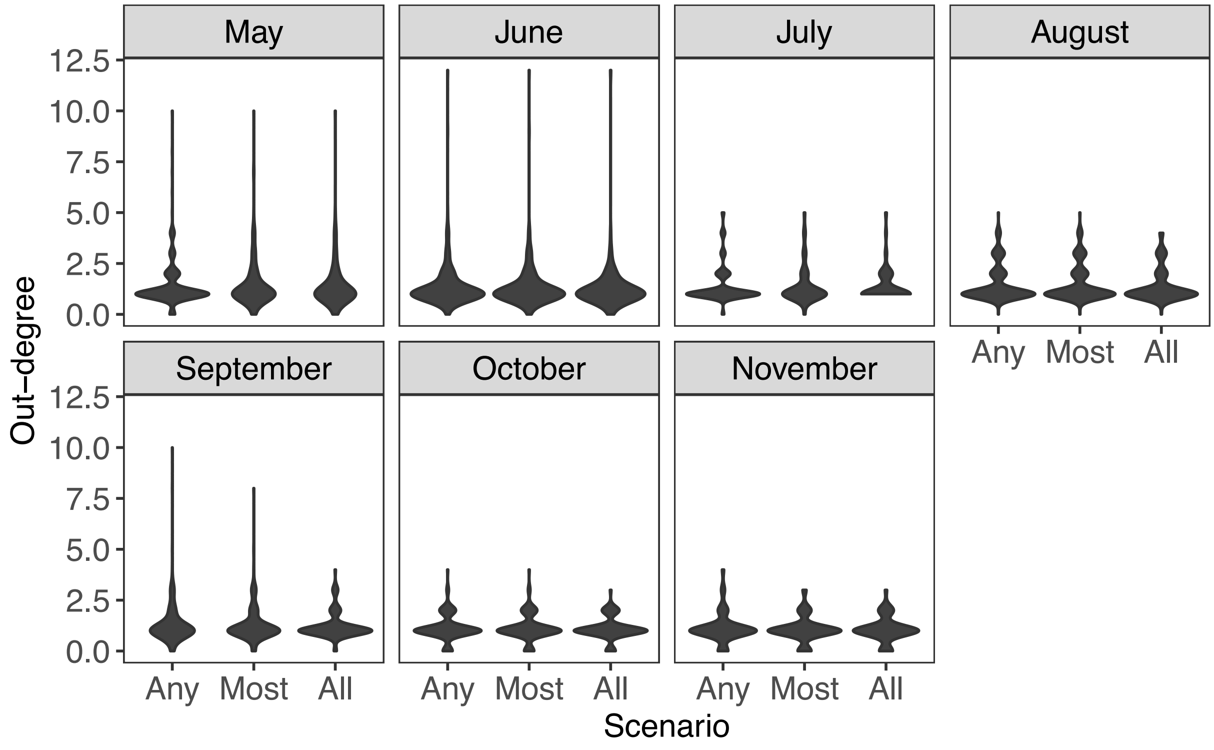

S5 Fig. Out-degree distribution for monthly horse movement networks in each data completeness scenario. Each violin plot represents a kernel density estimation of the out-degree of nodes included in each network. Scenarios: ‘any’ = at least one survey completed; ‘most’ = at least five surveys completed; ‘all’ = all seven surveys completed.

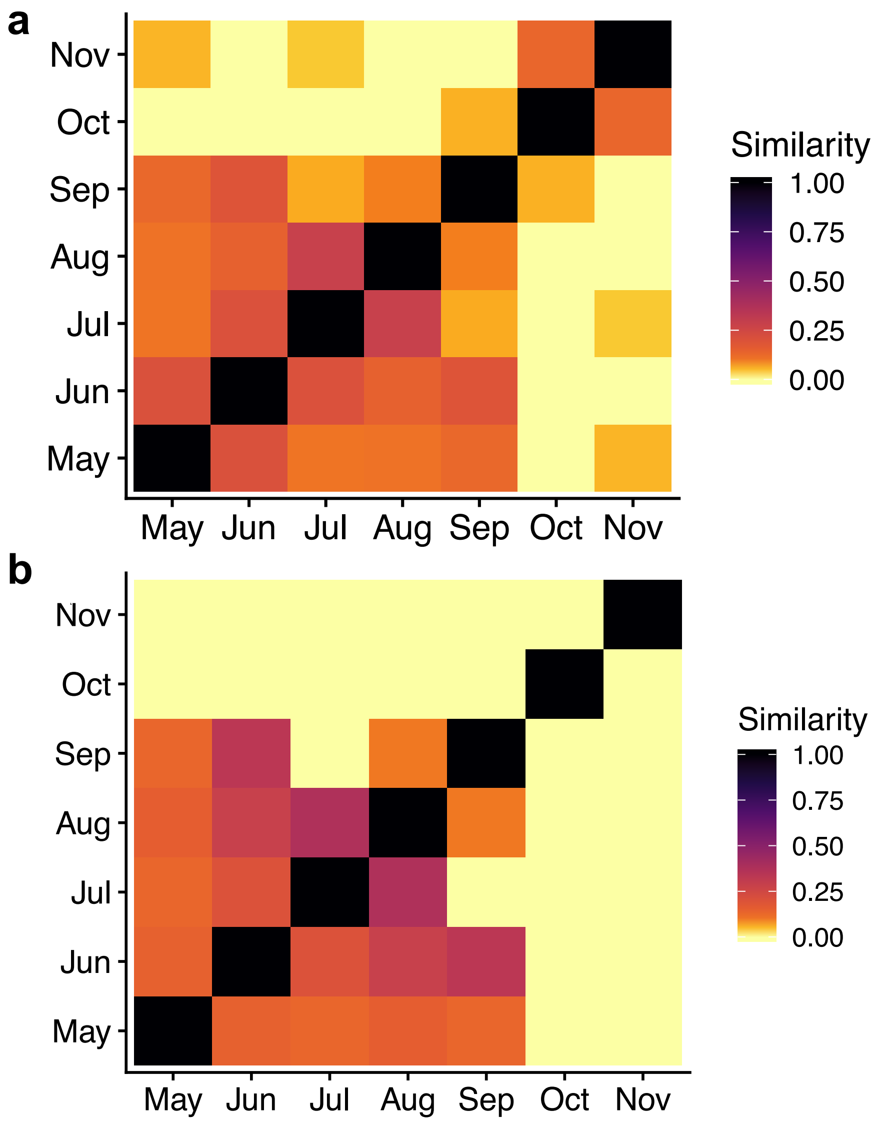

S6 Fig. The proportion of highly connected nodes that retained their position in consecutive monthly networks from May to November 2015. Highly connected nodes were defined as those within the top 10% of values according to their average degree. Panels represent the following data completeness scenarios: **(a)** at least one survey completed ('any'); **(b)** all surveys completed ('all').

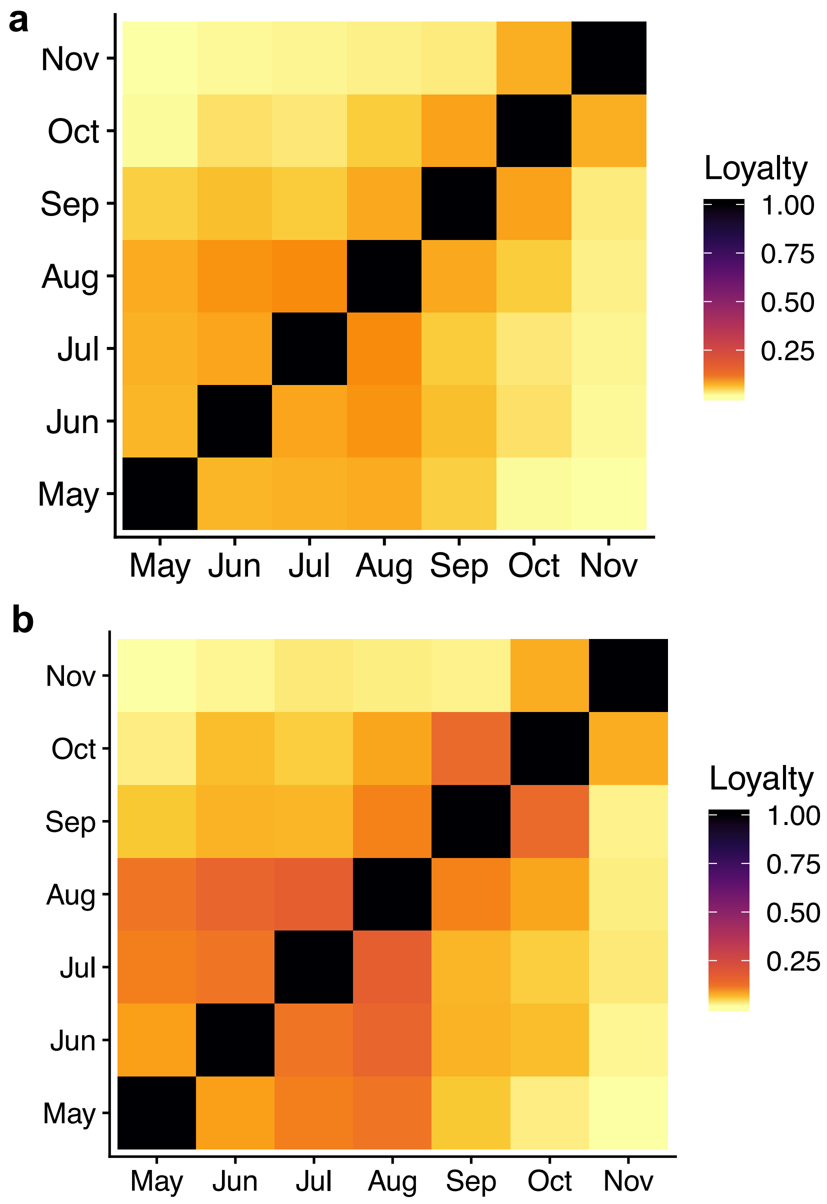

S7 Fig. The proportion of preserved direct contacts between locations in subsequent monthly networks from May to November 2015. Panels represent the following data completeness scenarios: **(a)** at least one survey completed ('any'); **(b)** all surveys completed ('all').
